# Preparation and Application of a Composite Formulation of Mint Essential Oil Nanoemulsion

**DOI:** 10.3390/insects17080776

**Published:** 2026-07-28

**Authors:** Lanmei Wei, Minting Liang, Xi Zeng, Wancheng Liu, Zhengwei Wu

**Affiliations:** 1 Department of Agronomy, College of Coastal Agricultural Sciences, Guangdong Ocean University, Zhanjiang 524088, China; 18289585376@163.com (L.W.); cherrysydney@163.com (M.L.); zx02120322@163.com (X.Z.); tomliu202408@163.com (W.L.); 2 South China Center, National Center of Technology Innovation for Saline-Alkali Tolerant Rice, Zhanjiang 524088, China

**Keywords:** mint essential oil, nanemulsion composite formulation, fall armyworm, corn aphid, green control

## Abstract

To develop an efficient and low-toxic green plant-based insecticide and address the issues of rapid volatilization and poor stability of mint essential oil, this study prepared a mint essential oil nanemulsion composite formulation using Tween 80 as the surfactant and propylene glycol as the cosurfactant. The insecticidal activity of the formulation was tested against fall armyworm, *Spodoptera frugiperda* and corn aphid, *Rhopalosiphum maidis*. Results showed that the optimal formulation consisted of a surfactant to cosurfactant mass ratio of 3:1 and a mixed surfactant to mint essential oil mass ratio of 9:1. The formulation exhibited good stability after dilution and centrifugation tests. Bioactivity assays indicated that the formulation effectively inhibited and killed fall armyworm eggs. At a concentration of 6.66 mg/mL, it completely inhibited the hatching of fall armyworm eggs and achieved 100% mortality of corn aphid nymphs and adults. This mint essential oil nanemulsion composite formulation demonstrated excellent performance and significant bioactivity, providing a new formulation option and technical support for the green control of fall armyworm and aphids on corn.

## Simple Summary

This study addressed the challenges of the volatile nature, poor stability, and low water solubility of mint essential oil, as well as the risk of resistance development and environmental pollution associated with chemical pesticides used for controlling corn pests. With the goal of developing highly effective and eco-friendly insecticides, nanoemulsion technology was employed to optimize the formulation, resulting in a mint essential oil nanoemulsion composite. The results showed that the optimal surfactant formulation ratio was Tween 80: propylene glycol 3:1 with a mixed surfactant-to-essential oil ratio of 9:1, yielding a preparation with small particle size and excellent stability under dilution and centrifugation conditions. At a concentration of 6.66 mg/mL, the preparation completely inhibited the hatching of fall armyworm, *Spodoptera frugiperda* eggs and achieved 100% mortality against both nymphs and adults of corn aphid, *Rhopalosiphum maidis*. This formulation provides a novel approach for green pest control in corn cultivation, reduces pesticide residues and the risk of resistance, and contributes to sustainable agricultural development.

## 1. Introduction

The fall armyworm *Spodoptera frugiperda* (Lepidoptera, Noctuidae) and the corn aphid *Rhopalosiphum maidis* (Hemiptera, Aphididae) are two highly fecund pests that commonly infest corn, severely compromising its yield and quality [1,2,3]. Traditional chemical pesticides have been the primary means of controlling these pests. However, their long-term use has led to pest resistance, environment pollution, and risks to human health. Therefore, there is an urgent need for novel, green, and safe control agents.

Mint essential oil, derived from mint plants, is known for its antibacterial and anti-inflammatory activities. It also exhibits contact-killing and repellent effects against pests and mites [4,5,6], positioning it as a plant-derived active substance with considerable development potential. For example, mint essential oil has been shown to possess insecticidal and repellent properties against aphids [7]. Fumigation experiments have demonstrated increasing mortality over time in *Bemisia tabaci*, *Trialeurodes vaporariorum* and *Tuta absoluta*, indicating significant lethal effects [8]. Additionally, it shows high toxicity to both eggs and adults of the two-spotted spider mite, along with oviposition and feeding deterrence against adults [9]. However, mint essential oil suffers from poor water solubility, high volatile, and instability, which greatly limits its bioavailability and practical field application.

Nanoemulsion technology enables the efficient dispersion of hydrophobic essential oils in the aqueous phase, resulting in a thermodynamically stable and optically transparent system. This significantly enhances the apparent water solubility and physicochemical stability of the essential oils. The particle size of nanoemulsions is typically below 200 nm, which increases the contact area with pest surfaces [10,11,12]. Boito et al. reported that a 5% cassia bark essential oil nanoemulsion exhibited nearly equivalent toxicity to flies as a 10% cassia bark essential oil [13]. Mahran developed a nanoemulsion using essential oils from various medicinal plants in Jizan, Saudi Arabia, as a green larvicidal agent for controlling *Culex* mosquitoes. The study showed weak insecticidal activity from cloves, safflower, and ginger oils, whereas nanoemulsions of basil and cumin demonstrated significant larvicidal efficacy [14]. Furthermore, research has shown that a 19.03% mint essential oil nanoemulsion can be used to control apple spider mites [15]. However, no studies have been published on the efficacy of mint essential oil nanoemulsions against fall armyworm or corn aphid.

Based on this, the present study employs nanemulsion technology using mint essential oil as the core active ingredient. The ratios of surfactants and co-surfactants are systematically screened and optimized to formulate mint essential oil nanoemulsion composites. These formulations are comprehensively evaluated in terms of appearance, particle size, and stability. Furthermore, the study investigates their inhibitory effects on the egg hatching of fall armyworm and their toxic effects on nymphs and adults of corn aphid. The aim is to provide an experimental basis and technical reference for the future field application in green pest management.

## 2. Materials and Methods

### 2.1. Materials

#### 2.1.1. Source of Insect Materials

The fall armyworm and corn aphid were collected from corn fields in Zhanjiang City and were used after being reared for multiple generations in an insect biochemical incubator in the laboratory. fall armyworm was reared at 28 °C, while corn aphid was reared at 26 °C, with a relative humidity of 65–70% and a photoperiod of 16:8 L:D. Fresh corn leaves were replaced daily. The corn leaves were obtained from pot-grown corn plants cultivated in a laboratory greenhouse.

#### 2.1.2. Experimental Materials

Mint essential oil was obtained from Huashuo Flavor & Fragrance Oil Co., Ltd. (Ji’an, China), brand Runshuo; Tween20, Tween40, Tween60, Tween80, Span80 from Sinopharm Chemical Reagent Co., Ltd. (Shanghai, China); propylene glycol from Jiuzhou CNOOC & Shell Petrochemical Co., Ltd. (Huizhou, China); polyglyceryl myristate from Weiyuchen Raw Material Store (Guangzhou, China); glycerol from Tianjin Chemical Reagent Co., Ltd. (Tianjin, China); chitosan oligosaccharide from Shanghai Aladdin Biochemical Technology Co., Ltd. (Shanghai, China); ultrapure water from Weihui City Shengyang Chemical Co., Ltd. (Xinxiang, China); 2% phosphotungstic acid solution from Feijing Biotechnology Co., Ltd. (Shanghai, China); absolute ethanol from Tianjin Zhiyuan Chemical Reagent Co., Ltd. (Tianjin, China).

#### 2.1.3. Instruments and Equipment

The UV–Vis spectrophotometer was obtained from Shanghai Youke Instrument Co., Ltd. Shanghai, China; the benchtop multipurpose high speed refrigerated centrifuge from Guangzhou Jidi Instrument Co., Ltd. Guangzhou, China; the dynamic light scattering (DLS) particle size analyzer from Malvern Instruments Ltd. Malvern, UK; the Zeta potential analyzer from Micromeritics Instrument (Shanghai) Co., Ltd. Shanghai, China; and the gas chromatography–mass spectrometry (GC–MS) system from Thermo Fisher Scientific Inc. Waltham, MA, USA.

#### 2.1.4. GC–MS Operating Conditions and Parameters

The chemical composition of the mint essential oil was identified using gas chromatography mass spectrometry (GC–MS). The mint essential oil was diluted with absolute ethanol at a ratio of 1:100 (*v*/*v*). The GC conditions were as follows: column, InertCap Pure–WAX (30 m length × 0.25 mm I.D. × 0.25 μm film thickness); injector temperature, 230 °C; oven temperature program, initially held at 40 °C for 2 min, then increased to 230 °C at a rate of 5 °C/min and held for 2 min; carrier gas, helium at a flow rate of 1.0 mL/min; injection mode, splitless. The MS conditions were as follows: ion source by electron impact (EI); ion source temperature 230 °C; interface temperature 230 °C; solvent delay 0.5 min; mass scan range *m*/*z* 33–600; ionization energy 70 eV.

### 2.2. Fabrication Process and Characterization Protocols of the Mint Essential Oil Nanoparticle Emulsion

#### 2.2.1. Surfactant Screening

Surfactants reduce interfacial tension and promote the spontaneous formation of microemulsions. Nonionic Tween surfactants are widely used in cosmetics and pharmaceuticals due to their environmental friendliness, good emulsifying properties, and cost effectiveness [16]. Among Tween 20, Tween 40, Tween 60, and Tween 80, Tween 80 was selected for having the highest HLB value (15) and strong hydrophilicity, which facilitates the dispersion of the oil phase in water2.2.2. Cosurfactant Screening.

Glycerol, Span 80, polyglycerol myristate, and propylene glycol were evaluated as cosurfactants. Each surfactant cosurfactant mixture was stirred until uniform, and those that formed clear, transparent, and stable solutions were selected as the preferred formulations.

#### 2.2.2. Determination of Km Value

The Km value, also referred to as the mixed surfactant ratio, represents the mixture of a surfactant and a cosurfactant. In this study, the selected surfactant and cosurfactant were prepared at mass ratios of 1:1, 2:1, 3:1, and 4:1. Each Km solution was then mixed with mint essential oil under stirring, while distilled water was added dropwise, and the resulting mixtures were recorded. The formulation that produced the largest region of nanoemulsion formation was selected.

#### 2.2.3. Preparation of Mint Essential Oil Nanoparticle Emulsion Composite

Referring to the microemulsion preparation method [17] developed by Ma Q et al. with appropriate modifications, the optimal ratio of surfactant, cosurfactant, and mint essential oil was screened using Origin 24.0 software to construct a pseudo-ternary phase diagram. The mint essential oil nanoemulsion composite formulation was then prepared via high energy emulsification. The optimized Km value of 3:1 was then combined with mint essential oil at a mass ratio of 9:1, stirred uniformly, and distilled water was added dropwise. Subsequently, chitosan oligosaccharides were introduced, and the mixture was stirred for 20–30 min using a magnetic stirrer until no particles remained and the emulsion became completely homogeneous. The resulting mixture was then homogenized for 20 min at 1000 rpm using an adjustable high-speed homogenizer, yielding a clear, transparent, non-layered, non-sedimented, and stable mint essential oil nanoemulsion composite formulation.

#### 2.2.4. Nanoemulsion Verification

When a laser beam passes through a colloid, if the diameter of the dispersed phase particles ranges between 1 and 100 nm (the defining characteristic of nanoemulsions), the beam is scattered by the particles, resulting in a clearly visible “light path” (Tyndall effect). To rapidly determine whether the prepared mint essential oil nanoemulsion composite formulation was at the nanoscale, and to preliminarily assess its stability and dispersion status, the formulation was irradiated with a laser pointer to observe whether the Tyndall effect was produced.

#### 2.2.5. Average Particle Size, PDI and Zeta Potential

After diluting the mint essential oil nanoemulsion composite preparation by 100-fold, the average particle size, polydispersity index (PDI), and zeta potential were measured using a laser particle size analyzer and a zeta potential analyzer.

### 2.3. Stability Study of Mint Essential Oil Nanoparticle Emulsion Composite Preparation

#### 2.3.1. Dilution Stability Test Protocols

The mint essential oil nanoemulsion composite preparation was diluted with distilled water at 1:20,1:40,1:60,1:80, and 1:100 ratios, and then made up to a final volume of 3 mL. After 24 h of static incubation, observations were made to determine whether any stratification or precipitation occurred.

#### 2.3.2. Centrifugal Treatment and Transmittance Calculation Protocol

Three mL of the mint essential oil nanoemulsion composite preparation was taken and centrifuged at 4000, 8000, and 10,000 rpm for 10 min. Observations were then made to determine whether stratification or precipitation occurred. When no stratification or precipitation was observed, ultrapure water was used as the control group. The absorbance of the mint essential oil nanoemulsion composite preparation was measured at 600 nm before and after centrifugation, and the transmittance was calculated using the following formula:

T = A/A_1_ × 100%

where T, A, and A_1_ denote transmittance, absorbance before centrifugation, and absorbance after centrifugation, respectively.

#### 2.3.3. Cold and Hot Exposure Conditions and Transmittance Assay

Three mL of the mint essential oil nanoemulsion composite preparation was placed into a 5 mL centrifuge tube and subjected to water baths at –20 °C, 4 °C, 25 °C, 40 °C, 60 °C, and 80 °C for 30 min each. Following each treatment, the appearance of the solution was examined for any changes, and purified water was used as a blank control, The absorbance of the mint essential oil nanoemulsion composite preparation was measured at 600 nm before and after heating, and its transmittance was calculated using the following formula:

T = A_2_/A_3_ × 100%

where A_2_ and A_3_ denote the absorbance before and after heating, respectively.

#### 2.3.4. Ambient Storage Conditions and Observation Protocol

Three mL of the mint essential oil nanoemulsion composite preparation was added to a 5 mL centrifuge tube and stored at room temperature for 30 days. Observations were subsequently made to determine whether stratification or precipitation occurred.

### 2.4. Inhibitory Effect of 3.3% Mint Essential Oil Nanoemulsion Composite Formulation on Egg Hatching of Fall Armyworm

The 3.3% mint essential oil nanoemulsion composite formulation was diluted with distilled water to prepare five different concentration gradients, corresponding to 0.17, 0.33, 0.67, 3.33, and 6.66 mL of the original formulation (final concentrations were adjusted accordingly with distilled water). Each concentration was tested with three replicates, and a blank control (distilled water) was included. For each treatment group and the control group, 15 eggs were used. Fresh, pale-green egg masses were collected from laboratory-reared fall armyworm females. Under a stereomicroscope, the egg masses were gently separated into individual eggs using a fine brush and insect pins. Healthy, undamaged eggs were selected and placed on the center of a filter paper. Subsequently, 100 µL of the corresponding concentration of the test solution was carefully dropped onto the egg surface using a micropipette to ensure complete coverage of each egg. After the liquid on the egg surface had air-dried naturally, the eggs were placed into ventilated 35 mL small rearing cages and kept in a constant-temperature incubator for observation (temperature 28 °C, relative humidity 65–70%, photoperiod 16:8 L:D). After 3–5 days, the number of hatched and unhatched eggs was recorded under a stereomicroscope. The egg hatching rate was calculated using the following formula:

Egg hatching rate (%) = (Number of hatched eggs/Total number of eggs) × 100.(1)

### 2.5. Insecticidal Activity of 3.3% Mint Essential Oil Nanoemulsion Against Corn Aphid Nymphs

Because the different instars of corn aphid nymphs are difficult to distinguish, and the nymphal stage lasts approximately 4–5 days with rapid instar transitions, the nymphs used in this experiment were not separated by instar. Instead, healthy nymphs of uniform size were selected. The 3.3% mint essential oil nanoemulsion composite formulation was diluted with distilled water to prepare five different concentration gradients, corresponding to 0.17, 0.33, 0.67, 3.33, and 6.66 mL of the original formulation (final concentrations were adjusted accordingly with distilled water). Each concentration was tested with three replicates, and a blank control (distilled water) was included. For each treatment group and the control group, 15 nymphs were placed into 35 mL small rearing cages, with corn leaves provided as the host plant. Prior to the experiment, the aphids were starved for 2 h. Subsequently, the plants and aphids in the treatment groups were sprayed with 0.5 mL of the corresponding test solution using a 30 mL small sprayer, while the control group was sprayed with 0.5 mL of distilled water. The aphids were then placed in an insect rearing chamber (temperature 26 °C, relative humidity 65–70%, photoperiod 16:8 L:D). After 24 h, the aphids were gently touched with a soft brush; those that did not move at all were considered dead.

### 2.6. Insecticidal Activity of 3.3% Mint Essential Oil Nanoemulsion Against Corn Aphid Adults

Healthy, apterous adult aphids of uniform size, approximately 1.8–2.2 mm in body length, were selected for the experiment. The 3.3% mint essential oil nanoemulsion composite formulation was diluted with distilled water to prepare five different concentration gradients, corresponding to 0.17, 0.33, 0.67, 3.33, and 6.66 mL of the original formulation (final concentrations were adjusted accordingly with distilled water). Each concentration was tested with three replicates, and a blank control (distilled water) was included. For each treatment group and the control group, 15 adult aphids were placed into 35 mL small rearing cages, with corn leaves provided as the host plant. Prior to the experiment, the aphids were starved for 2 h. Subsequently, the plants and aphids in the treatment groups were sprayed with 0.5 mL of the corresponding test solution using a small sprayer, while the control group was sprayed with 0.5 mL of distilled water. The aphids were then placed in an insect rearing chamber (temperature 26 °C, relative humidity 65–70%, photoperiod 16:8 L:D). After 24 h, the aphids were gently touched with a soft brush, those that did not move at all were considered dead.

### 2.7. Data Analysis

All experiments were repeated three times. Data were statistically analyzed using Excel 25.0. One-way analysis of variance (ANOVA) followed by Duncan’s new multiple range test for post hoc multiple comparisons was performed using SPSS 25.0 for the eggs of fall armyworm and the nymphs and adults of corn aphid. Bar charts were generated using Origin 24.0 with significance markers indicated in the graphs. Figure preparation was conducted using Adobe Illustrator 23.0. All results are presented as mean ± standard error.

## 3. Results

### 3.1. Chemical Composition of Mint Essential Oil Identified by GC–MS

The results of gas chromatography–mass spectrometry (GC–MS) analysis identified 41 volatile components in mint essential oil (Table 1). Quantitative analysis based on peak area normalization revealed that the main compounds and their relative contents were menthol and its stereoisomers, with a total relative content of approximately 24.07%, menthone (4.94%), and menthyl acetate (6.13%). These three compounds constituted the primary components of mint essential oil, with menthol being the main component.

### 3.2. Physicochemical Properties and Morphology of the Prepared Nanoparticle Emulsion Composite

#### 3.2.1. Screening Results of Surfactants

The molecular structure of Tween 80 contains both hydrophilic and hydrophobic groups, enabling it to reduce surface tension and exert emulsifying and dispersing effects in immiscible systems such as oil–water mixtures. Its hydrophilic–lipophilic balance (HLB) value is 15, and a higher HLB value indicates stronger hydrophilicity of the surfactant. For this reason, Tween 80 was selected as the surfactant.

#### 3.2.2. Screening Results of Auxiliary Surfactants

**Figure 1:**
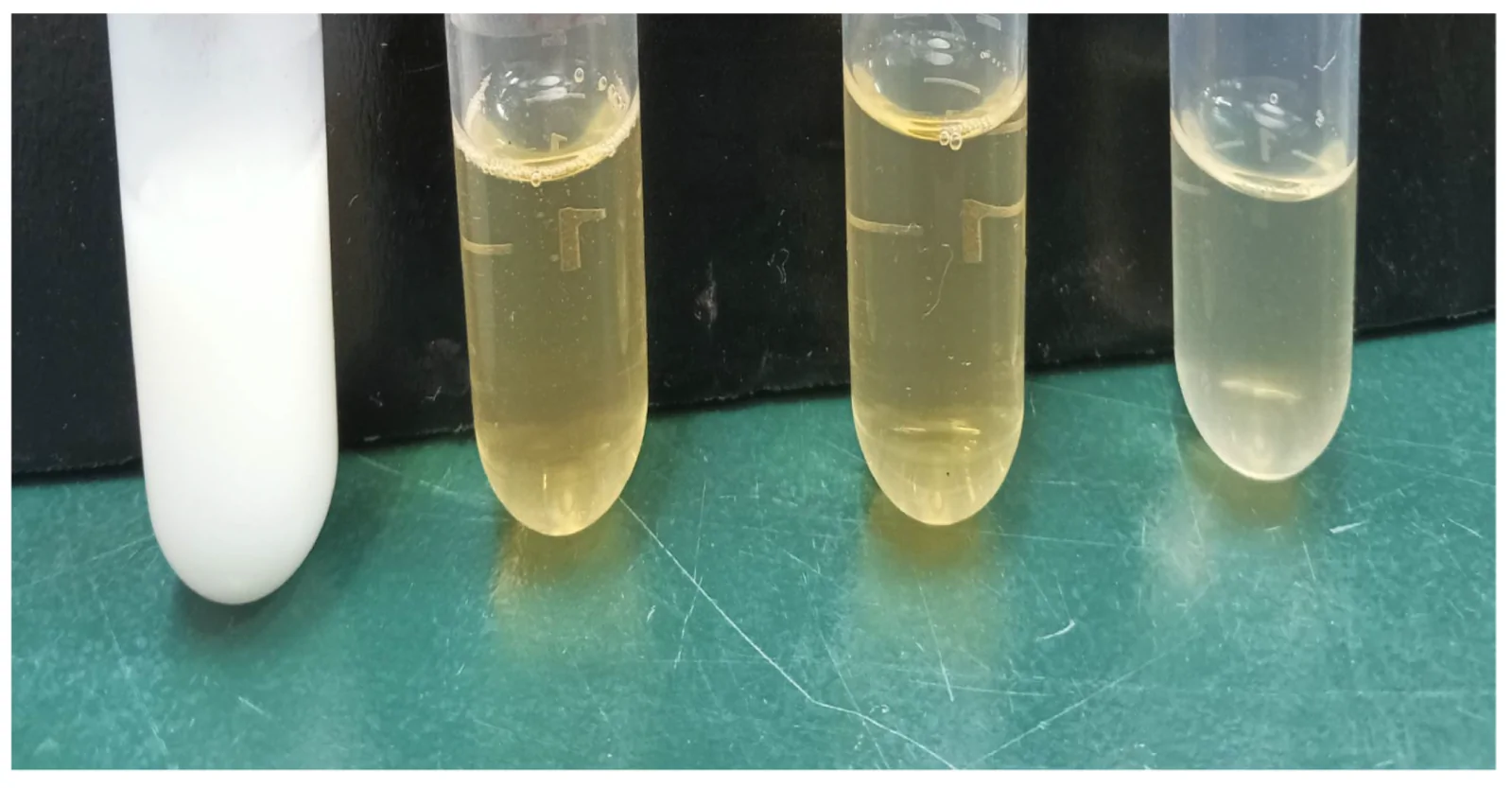
Appearance of liquids after mixing various surfactants with Tween 80. From left to right: Span, propylene glycol, glycerol, and polyglycerol myristate.

The appearance of the liquids formed by mixing different cosurfactants with Tween 80 varied. The emulsion prepared with Span 80 and Tween 80 appeared milky white, turbid, and opaque. Emulsions containing propylene glycol or glycerol with Tween 80 were yellow and translucent. The emulsion of polyglyceryl myristate with Tween 80 was pale yellow and translucent (Figure 1). Glycerol lacks hydrophobic groups and therefore cannot reduce interfacial tension or stabilize the dispersion system; consequently, it was not selected as a cosurfactant. Instead, propylene glycol and polyglyceryl myristate were chosen as candidate cosurfactants. In subsequent experiments, when polyglyceryl myristate was used as the cosurfactant, the emulsion appeared pale milky white, turbid, and opaque, with an average particle size of 131.0 nm and a polydispersity index (PDI) of 0.224. However, demulsification occurred after 3–5 days of storage. Based on these results, propylene glycol was ultimately selected as the cosurfactant.

#### 3.2.3. Result of Km Value Selection

**Figure 2:**
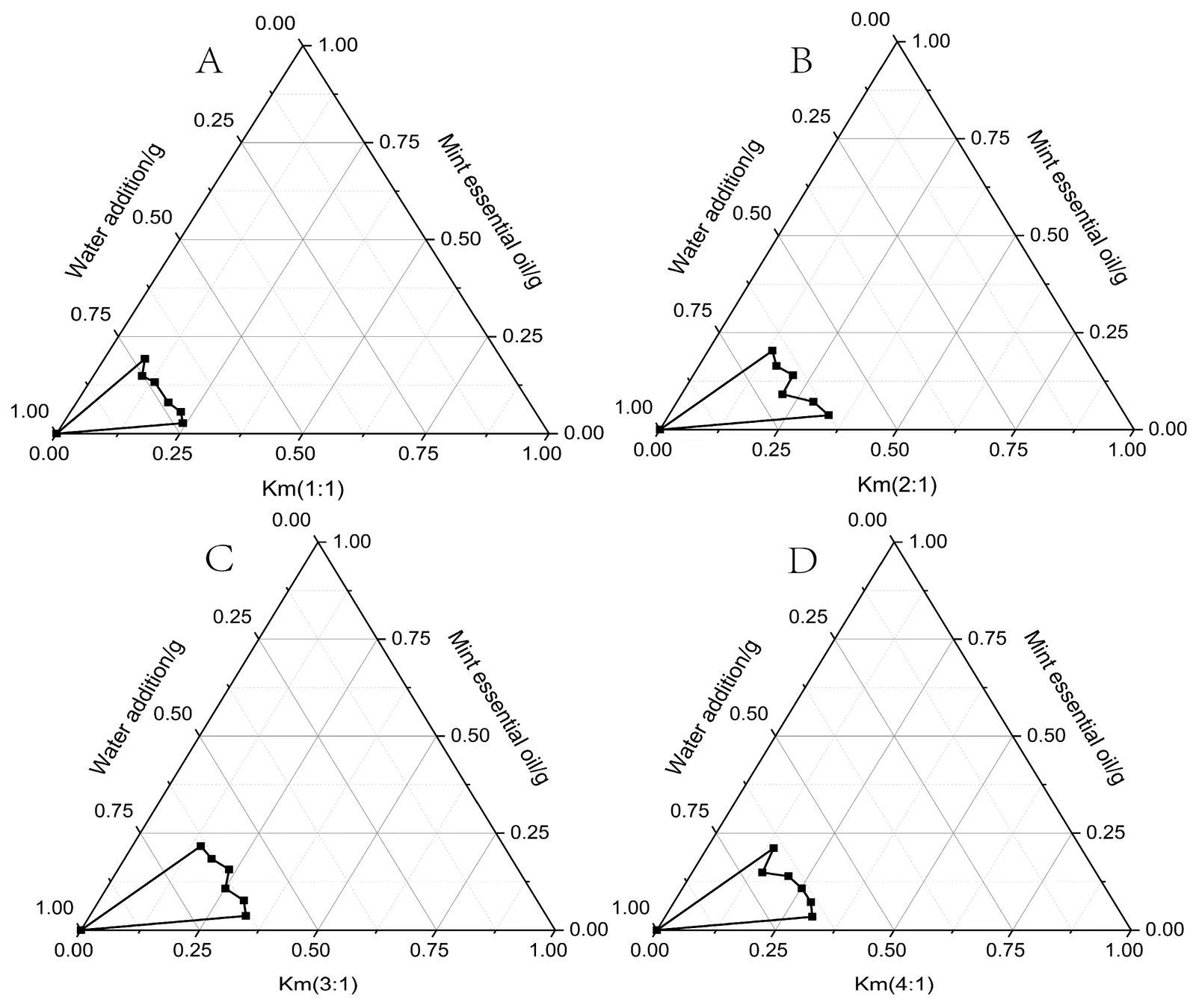
Pseudo-ternary phase diagrams of four mint essential oil nanoemulsion composite formulations prepared at different ratios (Km 1:1 (**A**), Km 2:1 (**B**), Km 3:1 (**C**), Km 4:1 (**D**)).

For the 1:1 Km value, six emulsion points were selected by mixing with mint essential oil at mass ratios of 9:1, 8:2, 7:3, 5:5, 4:6, and 3:7. Similarly, for Km values of 2:1, 3:1, and 4:1, six points were selected at mass ratios of 9:1, 8:2, 7:3, 6:4, 5:5, and 4:6. According to the pseudo-ternary phase diagram (Figure 2), the nanoemulsion region exhibited the largest area at a Km value of 3:1. Therefore, the optimal Km value for the mint essential oil nanoemulsion composite formulation was determined to be 3:1.

#### 3.2.4. Preparation of a Nanoemulsion Composite of Mint Essential Oil

The optimal Km value of 3:1 was selected and mixed with mint essential oil at mass ratios of 9:1, 8:2, 7:3, 6:4, 5:5, 4:6, 3:7, 2:8, and 1:9, followed by thorough stirring. The mixtures that appeared clear and transparent were selected. Among these, the nanoemulsion prepared with a Km value of 3:1 and a mint essential oil mass ratio of 9:1 exhibited the highest clarity, transparency, and stability (Table 2).

#### 3.2.5. Visual Observation and Tyndall Effect of the Nanoemulsion Composite

**Figure 3:**
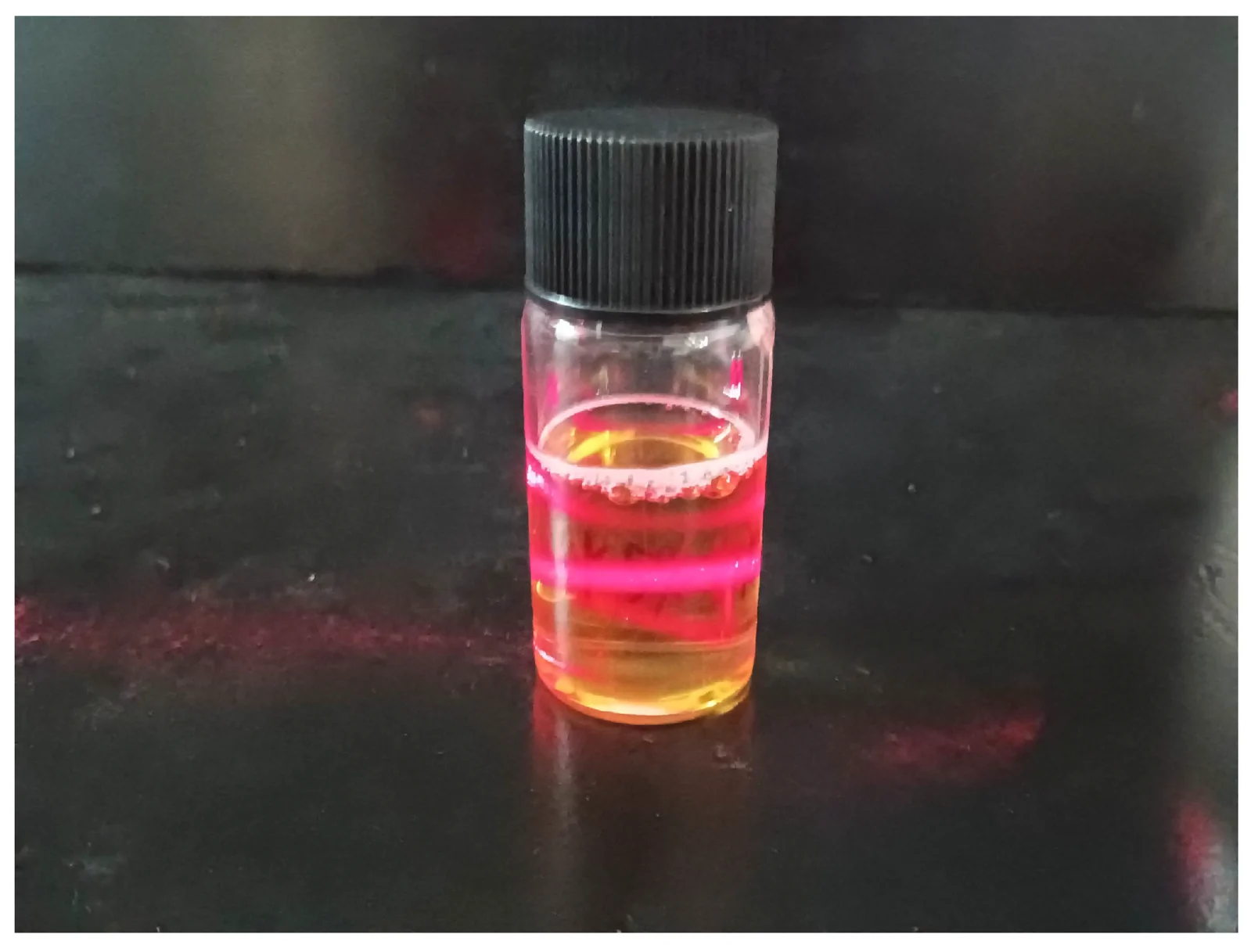
The Tyndall effect was observed in the mint essential oil nanoemulsion composite formulation.

When irradiated with a laser pointer, the mint essential oil nanoemulsion composite formulation exhibited a bright beam path within the emulsion, a clear indication of the Tyndall effect, which preliminarily confirmed the formation of the nanoemulsion. The prepared formulation appeared yellow, transparent, and clear, with no precipitation or stratification. Under laser pointer irradiation, the Darwin effect was observed (Figure 3).

#### 3.2.6. Average Particle Size, Polydispersity Index (PDI), and Zeta Potential

**Figure 4:**
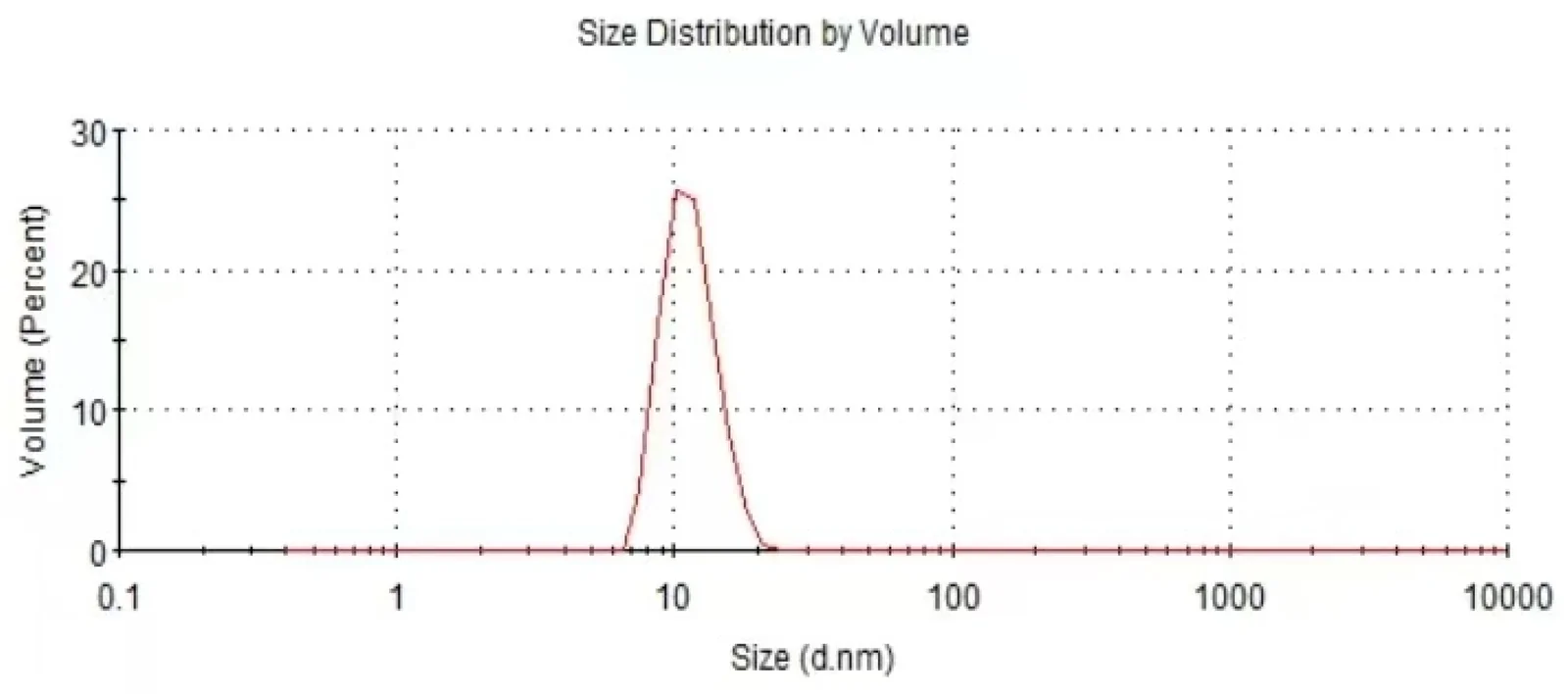
Particle size of the mint essential oil nanoemulsion composite preparation.

**Figure 5:**
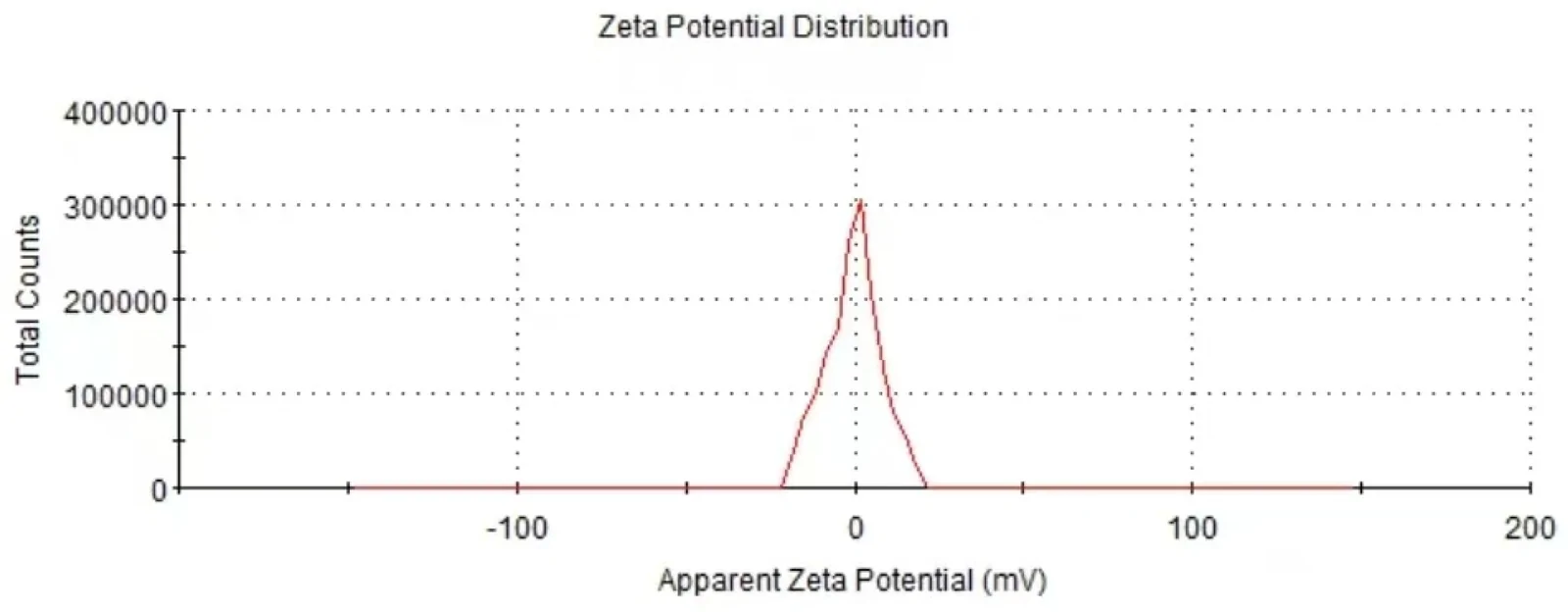
Zeta potential of the mint essential oil nanoemulsion composite formulation.

After at a ratio of 1:100, the particle size analysis prepared mint essential oil nanoemulsion composite formulation was diluted at a ratio of 1:100, particle size analysis using a nanolaser particle size analyzer showed an average particle size of 32.76 nm (Figure 4), a PDI of 0.51, and a Zeta potential of −0.2134 mV (Figure 5).

### 3.3. Stability of the Mint Essential Oil Nanoemulsion Composite Preparation

#### 3.3.1. Effect of Dilution on Nanoemulsion Stability

The prepared mint essential oil nanoemulsion composite was diluted with distilled water at a ratio of 1:20 to 1:100 and left undisturbed for 24 h. The emulsion remained transparent, clear, without stratification or sedimentation.

#### 3.3.2. Visual Appearance and Transmittance After Centrifugation

The prepared mint essential oil nanoemulsion composite formulation showed no sedimentation or stratification after centrifugation at 4000, 8000, and 10,000 r/min for 10 min. The calculated light transmittance values were 105.66%, 109.66%, and 102.20%, respectively, all exceeding 100%, indicating that the formulation possesses excellent centrifugal stability.

#### 3.3.3. Visual Appearance and Transmittance Across Temperature Gradients

The prepared mint essential oil nanoemulsion composite formulation showed no stratification or precipitation after thawing from −20 °C refrigeration, nor after exposure to 4 °C, 20 °C, 40 °C, and 60 °C. Upon heating to 80 °C, the formulation became more transparent. The measured light transmittance values at the respective temperatures were 92.97%, 92.78%, 107.92%, 93.70%, 96.66%, and 91.42%, all exceeding or approaching 100%. These results indicate that the mint essential oil nanoemulsion composite formulation possesses excellent thermal stability.

#### 3.3.4. Visual Observations After 30-Days Ambient Storage

The prepared mint essential oil nanoemulsion composite formulation was stored at room temperature indoors. After 30 days, the formulation showed no visible stratification or sedimentation, indicating excellent storage stability.

### 3.4. Inhibition of Fall Armyworm Egg Hatching

**Figure 6:**
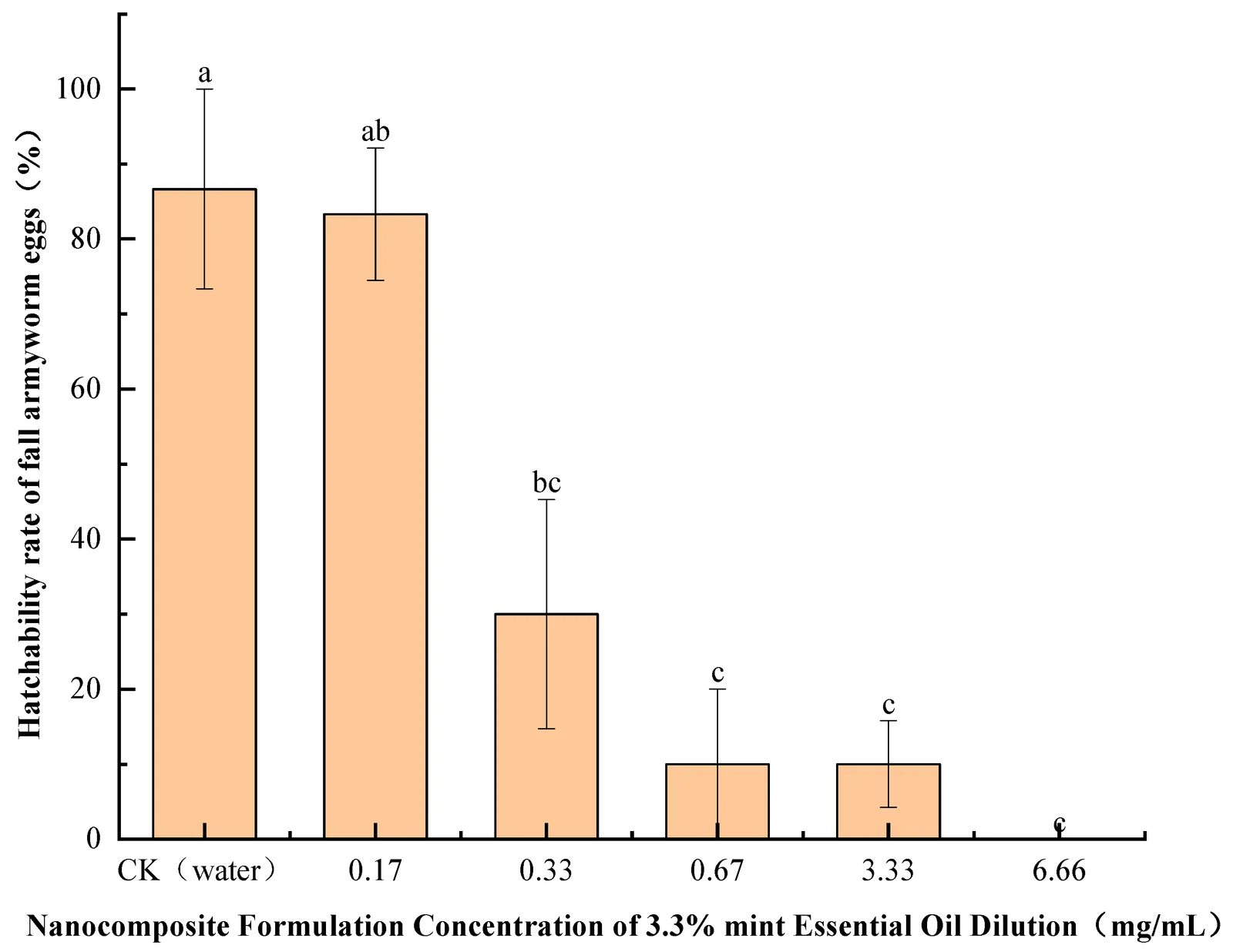
Insecticidal activity of 3.3% mint essential oil nanoemulsion composite formulation at various concentrations on eggs of fall armyworm. Treatments with bars with different letters are statistically different from each other (*p* < 0.05). Error bars represent standard error (SE) of the mean.

**Figure 7:**
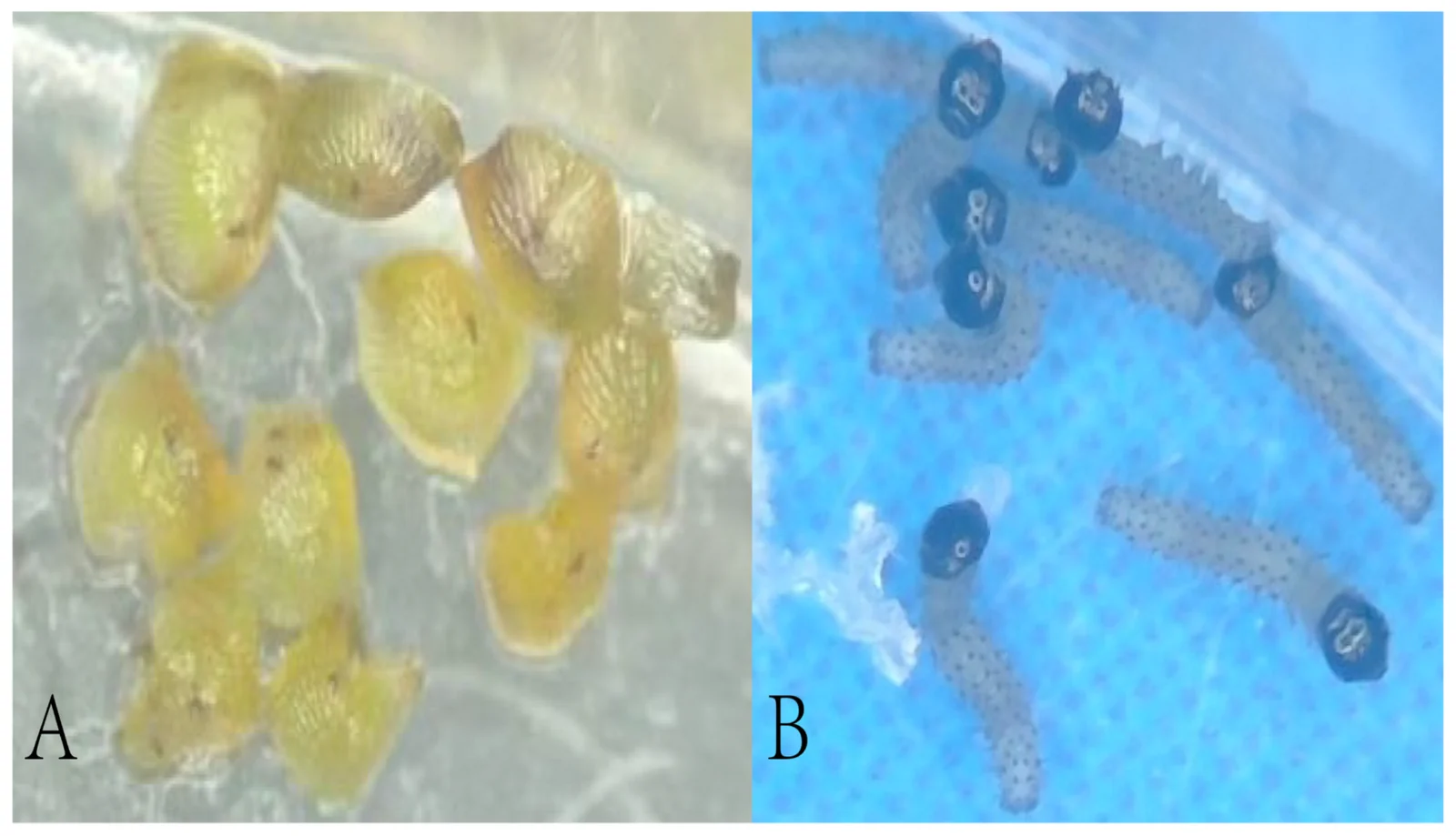
Morphology of unhatched (**A**) and hatched (**B**) eggs of fall armyworm.

The effects of different concentrations of the 3.3% mint essential oil nanoemulsion composite formulation on the egg hatching of fall armyworm varied. As the concentration increased, the inhibitory effect of the formulation became more pronounced. The egg hatching rates in the control group (0 mg/mL) and the 0.17 mg/mL treatment group were 86.67 ± 13.33% and 83.33 ± 8.82%, respectively, with no significant difference between them. In contrast, all other concentrations showed statistically significant differences compared to the control group. Notably, at a concentration of 6.66 mg/mL, the egg hatching rate was 0% (Figure 6), demonstrating the formulation’s capacity to inhibit fall armyworm egg hatching. A visual comparison between unhatched and hatched eggs of fall armyworm is shown in Figure 7.

### 3.5. Toxicity to Corn Aphid Nymphs

**Figure 8:**
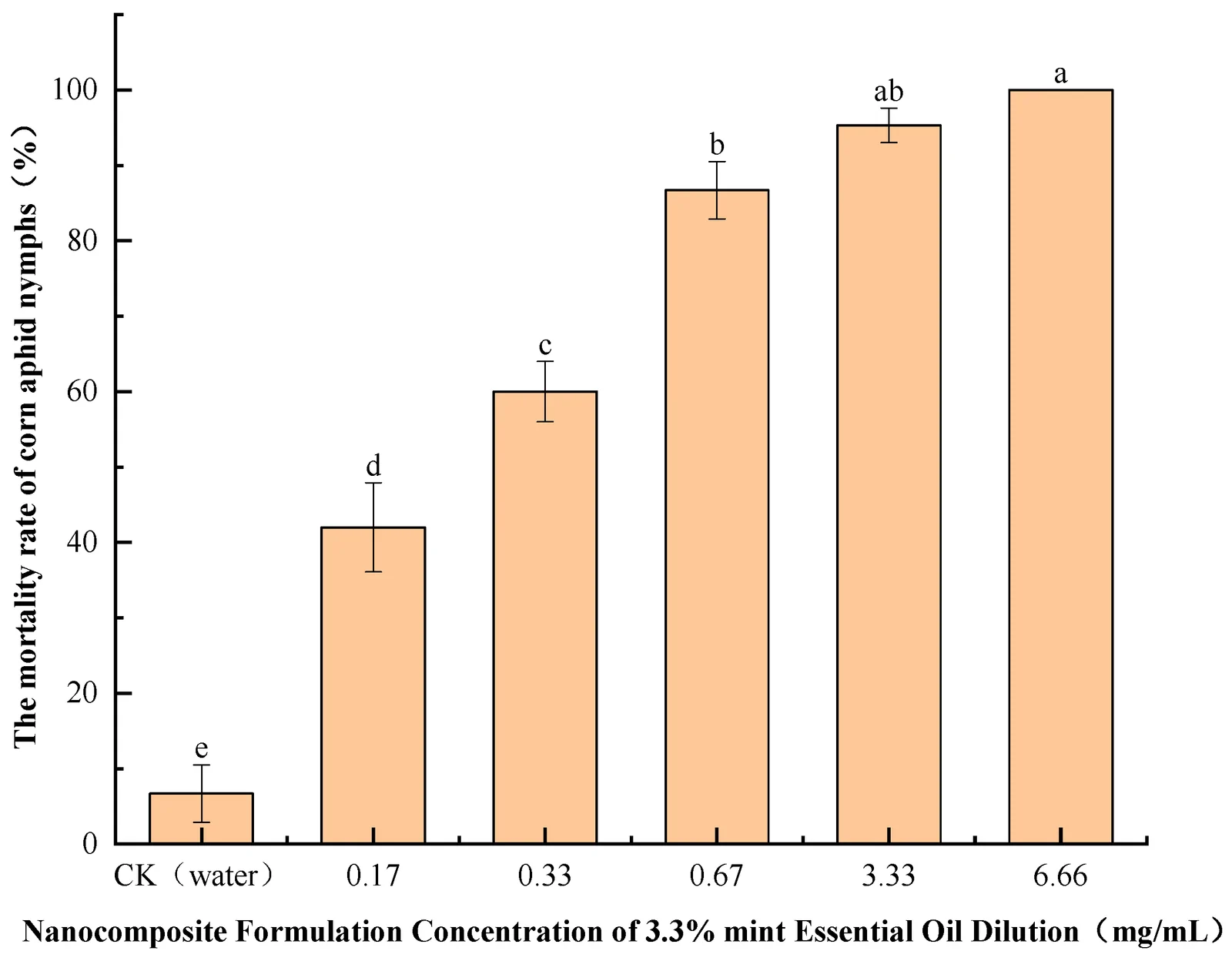
Insecticidal activity of 3.3% mint essential oil nanoemulsion composite formulations at various concentrations against corn aphid nymphs. Treatments with bars with different letters are statistically different from each other (*p* < 0.05). Error bars represent standard error (SE) of the mean.

**Figure 9:**
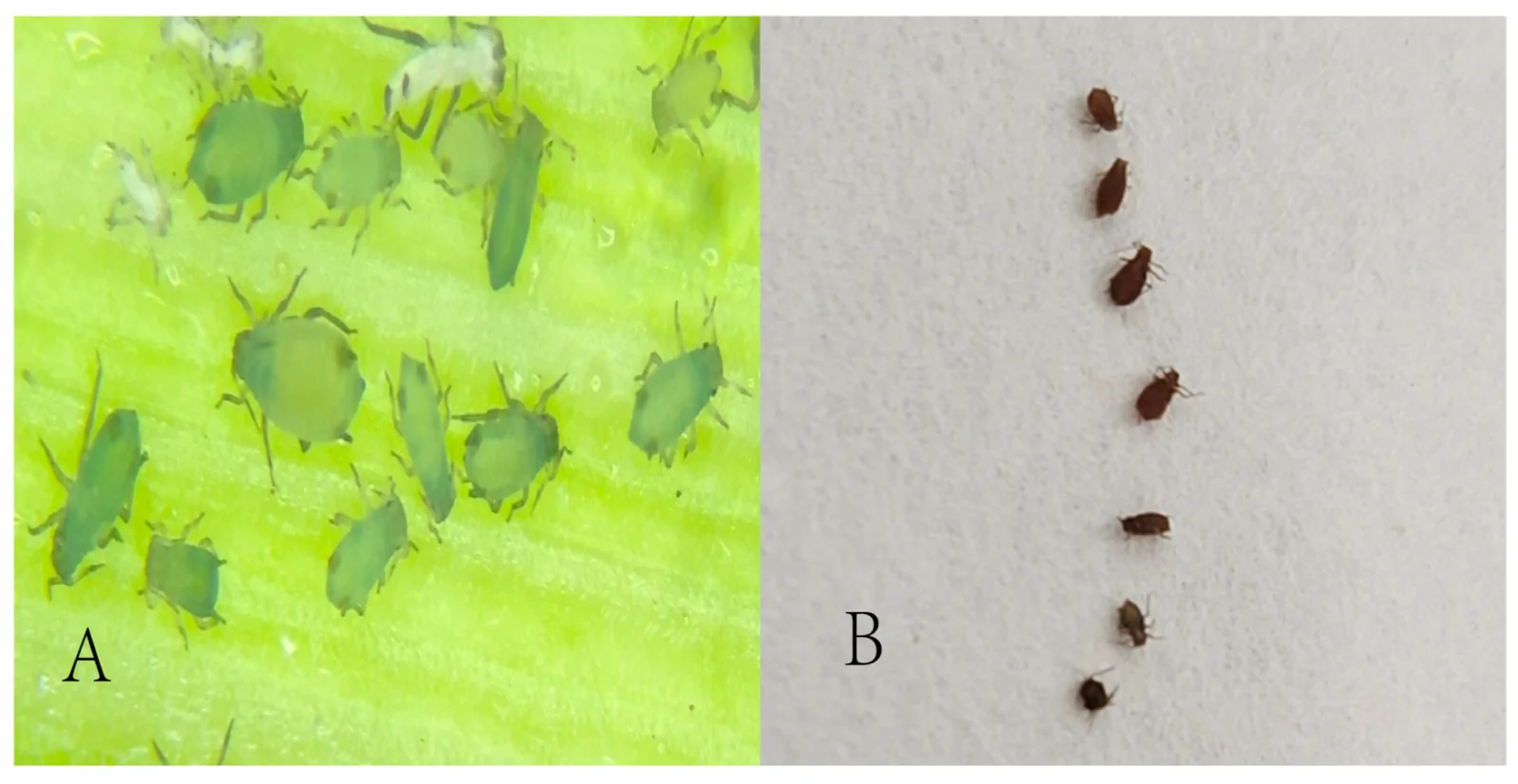
Morphology of normal (**A**) and dead (**B**) corn aphid nymphs.

As the dilution concentration increased progressively, the toxic efficacy of the 3.3% mint essential oil nanoemulsion composite formulation against corn aphid nymphs gradually enhanced. The mortality rate of corn aphid nymphs was 6.7 ± 3.8% at a concentration of 0 mg/mL (control group) and increased to 42 ± 5.9% at 0.17 mg/mL, showing a significant difference compared to the control. Notably, at a concentration of 6.66 mg/mL, the mortality rate reached 100%, a statistically significant value (*p* < 0.005) (Figure 8). After treatment with the mint essential oil nanoemulsion composite formulation, the normally green corn aphid nymphs displayed darkened or blackened bodies that became curled and rigid upon death (Figure 9).

### 3.6. Toxicity to Corn Aphid Adults

**Figure 10:**
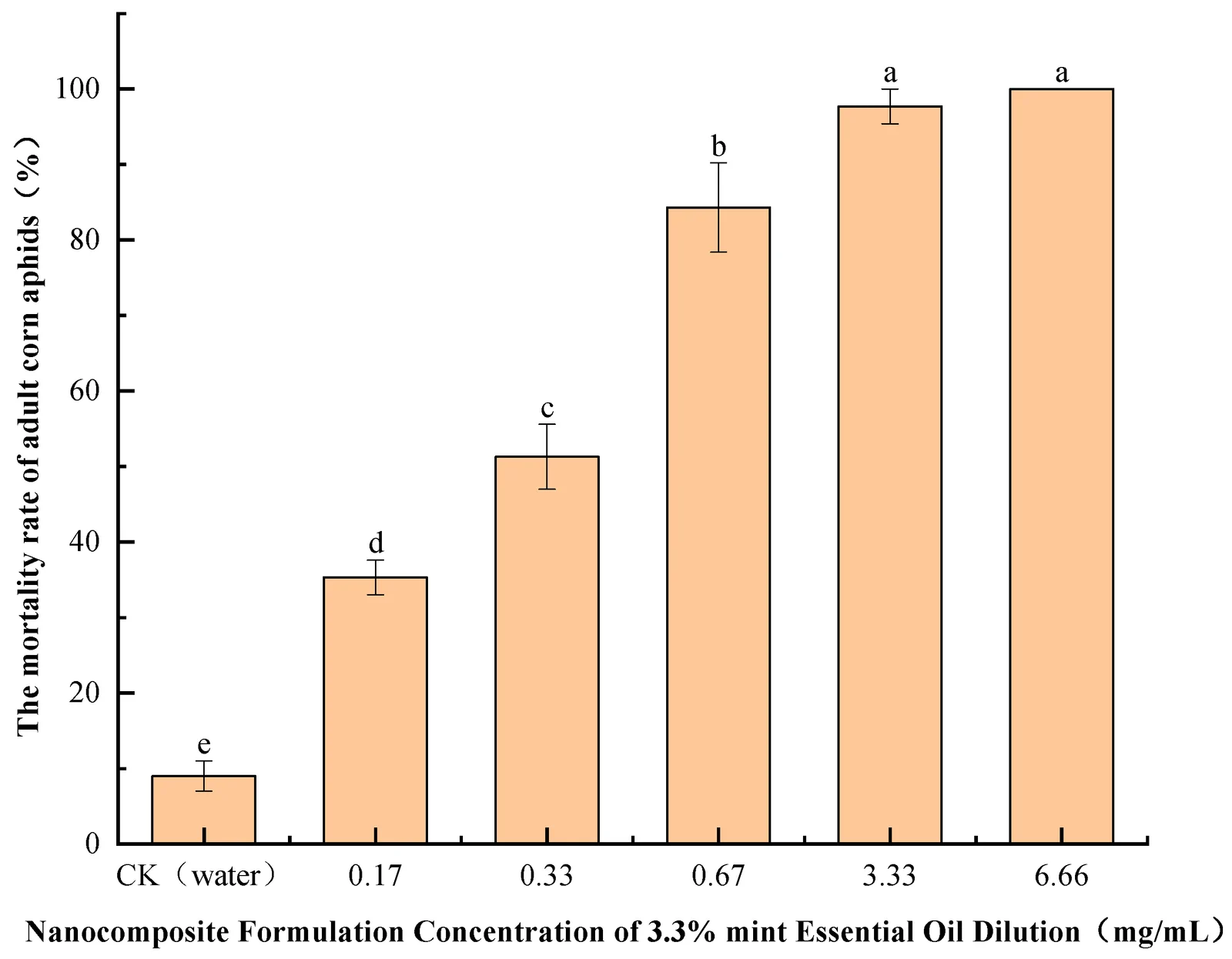
Insecticidal activity of 3.3% mint essential oil nanoemulsion composite formulations at various concentrations against corn aphid adults. Treatments with bars with different letters are statistically different from each other (*p* < 0.05). Error bars represent standard error (SE) of the mean.

As the dilution concentration gradually increased, the 3.3% mint essential oil nanoemulsion composite formulation progressively exhibited an enhanced toxicity on adult corn aphids. In the control group (0 mg/mL), the mortality rate of adult corn aphids was 9% ± 2% whereas at concentrations of 0.17 mg/mL and 0.33 mg/mL, the mortality rates increased to 35.3% ± 2.3% and 51.3% ± 4.3%, respectively. At higher concentrations, mortality rates exceeded 80%, and the 6.66 mg/mL formulation achieved a 100% mortality rate. All treatment groups showed statistically significant differences compared to the control group (Figure 10).

### 3.7. Nymph Versus Adult Mortality in Corn Aphids

**Figure 11:**
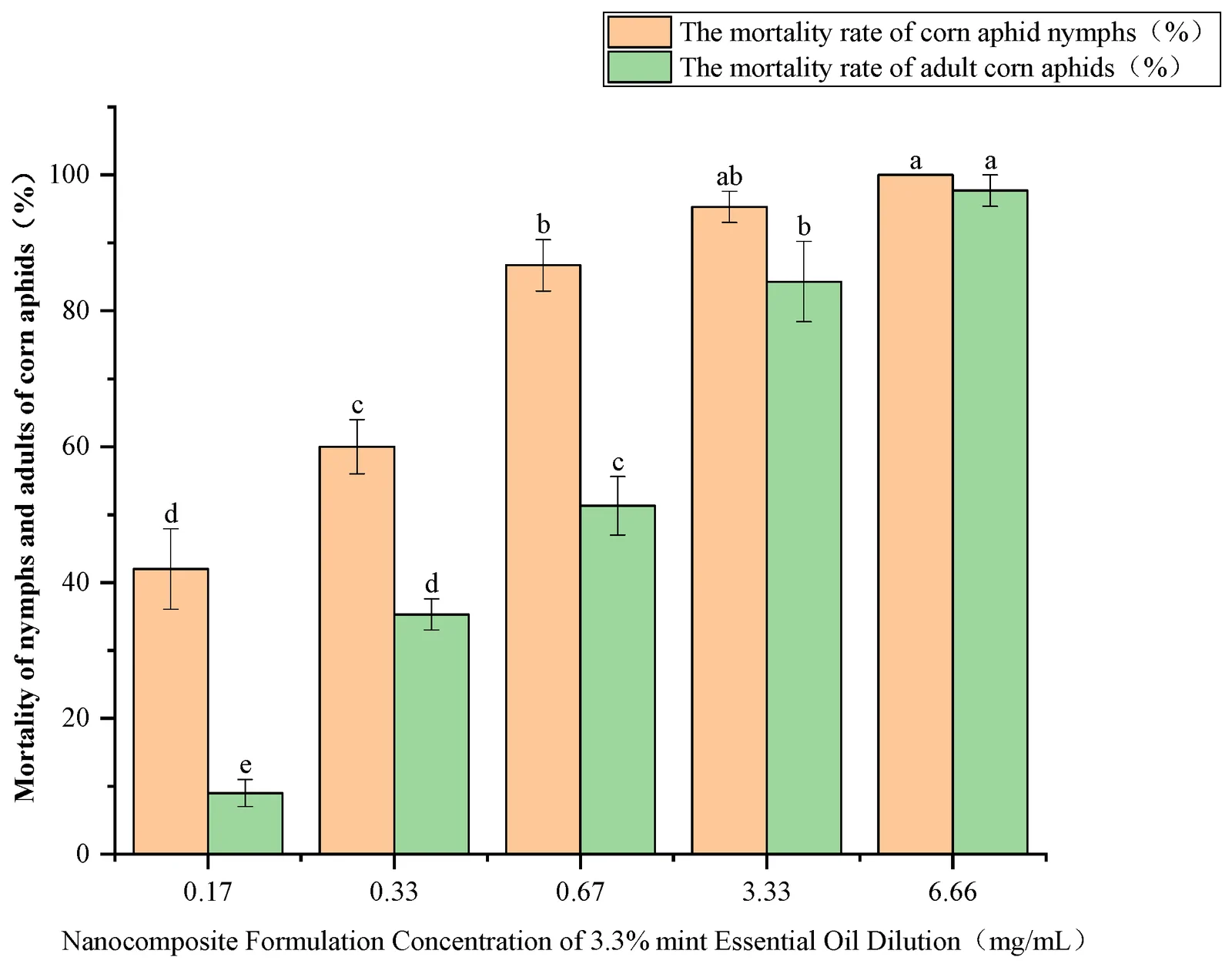
Mortality rate of nymphs and adults of corn aphids induced by different concentrations of the 3% mint Essential Oil Nanoemulsion Composite Formulation. Treatments with bars with different letters are statistically different from each other (*p* < 0.05). Error bars represent standard error (SE) of the mean.

As shown in Figure 11, significant differences were observed in the mortality of nymphs and adults of corn aphids at 0.17, 0.33, and 0.67 mg/mL concentrations, while no significant differences were detected at 3.33 and 6.66 mg/mL concentrations.

## 4. Discussion

Plant essential oils exhibit a range of biological activities against agricultural pests, including feeding inhibition, toxicity, and repulsion, highlighting their potential for the development of green formulations [15,18,19]. For instance, essential oils derived from pepper, chili, and neem have been shown to significantly inhibit feeding and reduce oviposition in fall armyworm [16,20]. Nevertheless, most studies have focused exclusively on larval or adult stages, with limited reports addressing efficacy during the egg stage. Furthermore, conventional essential oil formulations often suffer from poor water solubility and high volatility, posing substantial challenges to meeting the demands of large-scale application.

Currently, two major pests in corn fields the fall armyworm and corn aphids are primarily controlled using chemical pesticides, such as chlorantraniliprole [21], emamectin benzoate–chlorfenapyr [22], flufenoxuron [23], and imidacloprid [24,25,26,27]. However, the prolonged and repeated use of chemical pesticides can easily lead to pest resistance and raise environmental safety concerns. Therefore, there is an urgent need to develop safe, efficient, and green alternative control technologies [11,14].

This study employed nanoemulsion technology based on safe raw materials, a simple process, and environmental friendliness. Tween 80 was used as the primary surfactant, with propylene glycol as the cosurfactant, as a single surfactant was found to be insufficient for emulsifying mint essential oil successfully. The optimal formulation (Km = 3:1; ratio of mixed surfactants to mint essential oil = 9:1) was determined using a pseudo-ternary phase diagram, leading to the successful production of a stable and transparent mint essential oil nanoemulsion. The resulting preparation exhibited an average particle size of 32.76 nm and excellent dispersibility, maintaining stability under conditions of dilution, centrifugation, temperature stress, and room temperature storage for 30 days. This approach effectively addresses the technical challenges associated with the poor water solubility, high volatility, and insufficient stability of mint essential oil [11,28,29,30].

Bioactivity assay results showed that the inhibitory effect of the mint essential oil nanoemulsion composite formulation on the hatching of fall armyworm eggs, as well as its toxic efficacy against nymph and adult corn aphids, increased with rising concentration. Its pest control performance was significantly superior to that of conventional essential oil formulations [20,31,32], demonstrating the possibility to manage two major corn pests simultaneously. Compared with existing studies both domestically and internationally, this formulation features smaller particle sizes, more uniform dispersion, and enhanced stability under thermal and dilution conditions [33,34,35], outperforming reported systems such as eugenol essential oil nanoemulsions and conventional mint essential oil nanoemulsions [31]. This study confirms that the mint essential oil nanoemulsion composite formulation is capable of controlling fall armyworm (at the egg stage) and nymph and adult corn aphids, thereby achieving dual pest control with simultaneous egg eradication a breakthrough that fills a research gap in this field [32,36,37,38]. Nevertheless, limitations of this study include a narrow target pest spectrum, insufficient rapid and sustained efficacy, and a concentration-dependent effect.

This study offers novel formulation options derived from plants and provides key technical support for the green control of fall armyworm and corn aphids. The preparation methods and optimization strategies for formulation ratios presented herein can serve as a reference for the development of nanoemulsion formulations using other plant-derived essential oil pesticides.

Future research should include field efficacy trials of this composite formulation to determine the optimal application concentration, application method, and duration of residual efficacy. Additionally, its safety profile with respect to non-target organisms should be evaluated, so as to provide more comprehensive experimental evidence for its widespread adoption.

## 5. Conclusions

In summary, this study prepared a mint essential oil nanoemulsion composite using Tween 80 as the surfactant and propylene glycol as the cosurfactant. The results indicated that the optimal formulation consisted of a surfactant-to-co-surfactant mass ratio of 3:1 and a mixed-surfactant-to-mint-essential oil mass ratio of 9:1. The resulting nanoemulsion composite exhibited toxic and inhibitory effects against fall armyworm eggs and corn aphids, with biological activity increasing in a concentration-dependent manner.

## Figures and Tables

**Table 1:** GC–MS Analysis of Volatile Components in mint Essential Oil.

No.	Retention Time (min)	Compound Name	Relative Content (%)
1	7.135	α-Pinene	0.48
2	7.192	α-Pinene	0.33
3	7.595	trans-1-Methyl-4-(1-methylethyl)cyclohexane	1.25
4	7.653	γ-Terpinene	0.70
5	8.064	4-Methyl-1-(1-methylethyl)cyclohexene	0.18
6	8.303	p-Menth-3-ene	0.22
7	8.882	α-Fenchene	2.75
8	9.134	α-Fenchene	2.07
9	9.295	Sabinene	0.99
10	9.845	β-Pinene	0.61
11	10.069	β-Myrcene	1.98
12	10.488	α-Terpinene	0.50
13	11.247	3-Isopropenyl-5,5-dimethylcyclopentene	8.04
14	11.885	γ-Terpinene	1.06
15	12.368	1,3,8-p-Menthatriene	1.44
16	12.673	(+)-4-Carene	1.47
17	13.854	1-Hexanol	0.48
18	14.860	3-Octanol	1.60
19	15.452	3-(4-Methyl-3-pentenyl)furan	0.22
20	15.871	1-Methyl-3-(1-methylethenyl)benzene	0.62
21	16.104	3-Methylcyclohexanol	0.64
22	17.070	cis-5-Methyl-2-(1-methylethyl)cyclohexanone	8.10
23	17.593	Menthone	4.94
24	17.901	Camphor	0.44
25	18.172	Dihydroumbellulone	0.75
26	19.093	Menthyl acetate	6.13
27	19.494	dl-Menthol	5.69
28	20.872	(±)-Menthol	18.38
29	21.685	α-Terpineol	2.62
30	22.099	Viridiflorene	0.25
31	22.601	2-Isopropyl-5-methyl-3-cyclohexen-1-one	1.92
32	22.938	ε-Amorphene	0.82
33	23.392	δ-Cadinene	0.58
34	23.801	Myrtenol	0.22
35	24.596	Anethole	0.46
36	25.468	(E)-6,10-Dimethyl-5,9-undecadien-2-one	0.25
37	27.081	Piperitenone	0.09
38	28.549	8,9-Dehydrothymol	0.27
39	30.045	(E)-Nerolidol	0.06
40	32.693	3-Allyl-6-methoxyphenol	0.04
41	33.132	Thymol	0.06

**Table 2:** Optimal Km values and water ratios for mint essential oil.

Mass Ratio	Mint Essential Oil/g	Mixed Surfactant/g	Water Addition/g	Experimental Phenomena
1:9	0.10	0.90	1.73	The emulsion appears uniform, translucent, and yellow in color.
2:8	0.20	0.80	1.62	The emulsion appears uniform, semi-translucent, and pale yellow in color.
3:7	0.30	0.70	1.79	The emulsion appears uniform, non-shiny, and slightly yellow in color.
4:6	0.40	0.60	1.56	The emulsion appears uniform, opaque, and slightly yellow in color.
5:5	0.50	0.50	1.72	The emulsion appears turbid, opaque, and pale milky white in color.
6:4	0.60	0.40	1.77	The emulsion appears turbid, opaque, and pale milky white in color.
7:3	0.70	0.30	1.52	The emulsion is unstable with oil–water separation.
8:2	0.80	0.20	1.54	The emulsion is unstable with oil–water separation.
9:1	0.90	0.10	1.79	The emulsion is unstable with oil–water separation.

## Data Availability

Data is available on request.
